# A Green’s Function Molecular Dynamics Approach to the Mechanical Contact between Thin Elastic Sheets and Randomly Rough Surfaces

**DOI:** 10.3390/biomimetics1010007

**Published:** 2016-10-27

**Authors:** Carmine Putignano, Wolf B. Dapp, Martin H. Müser

**Affiliations:** 1 Department of Mechanical Engineering, Imperial College London, Exhibition Road, South Kensington, London SW7 2AZ, UK; 2 Department of Mechanics, Mathematics and Management, Polytechnic University of Bari, Bari 70126, Italy; 3 Forschungszentrum Jülich, John von Neumann Institut für Computing and Jülich Supercomputing Centre, Institute for Advanced Simulation, 52425 Jülich, Germany; w.dapp@fz-juelich.de; 4 Department of Materials Science and Engineering, Saarland University, 66123 Saarbrücken, Germany; martin.mueser@mx.uni-saarland.de

**Keywords:** rough contact mechanics, thin layers, contact area

## Abstract

Adhesion of biological systems is often made possible through thin elastic layers, such as human skin. To address the question of when a layer is sufficiently thin to become adhesive, we extended Green’s function molecular dynamics (GFMD) to account for the finite thickness of an elastic body that is supported by a fluid foundation. We observed that thin layers can much better accommodate rough counterfaces than thick structures. As a result, the contact area is enlarged, in particular, when the width of the layer *w* approaches or even falls below the short-wavelength cutoff $\lambda_{s}$ of the surface spectra. In the latter case, the proportionality coefficient between area and load scales is ${\left(w/\lambda_{s}\right)}^{3}$, which is consistent with Persson’s contact mechanics theory.

## 1. Introduction

A central aspect in many engineering applications is the mechanical contact between nominally flat but microscopically rough surfaces. It affects important issues such as friction and wear between solids, energy efficiency, and durability as well as environmental compatibility of many devices. Much progress has been made on the topic in the past few decades. Many analytical, numerical, and experimental approaches have been pursued to determine what occurs, from the macro- to the microscales, when two surfaces come into contact. Most of the attention so far has been focused on surfaces resting on bulk solids, and generally described with a semi-infinite. This is in contrast to many biological surfaces, which often have a hierarchical structure like gecko’s feet or consist of thin, elastic layers resting on a fluid-like foundation, such as human skin.

**Figure 1:**
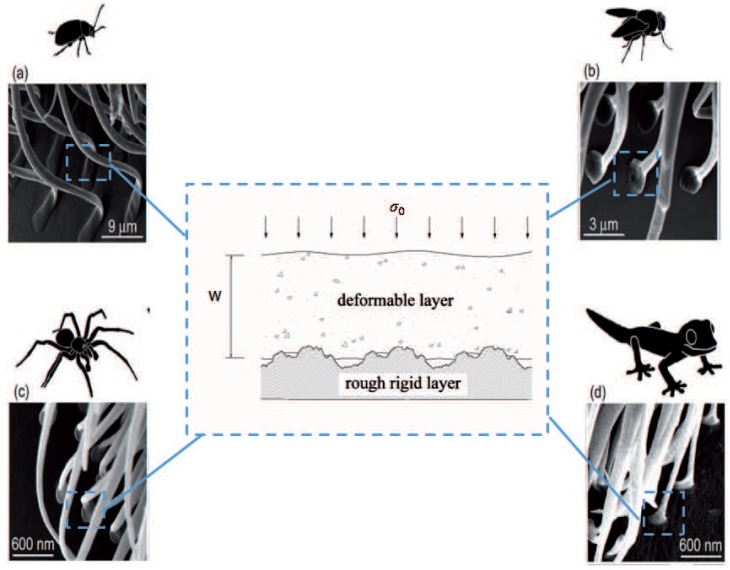
Natural examples of spatula-shaped terminal elements : (**a**) beetle; (**b**) fly; (**c**) spider; (**d**) Tokay gecko. Cryo-scanning electron microscopy (cryo-SEM) images adapted from [8] - Reproduced by permission of The Royal Society of Chemistry. All the different cases are reduced to the model in the inset where a rough rigid surface is in contact with an elastic layer supported by a constant pressure ($\sigma_{0}$).

An analysis of the contact mechanics of thin layers may thus constitute a first step towards a better understanding of adhesion in biological systems. Indeed, adhesive devices reproducing, on an industrial scale, the amazing adhesive properties of natural systems, are much sought-after. Examples for the latter span a wide range of scales [1,2,3,4,5,6,7,8,9] from insects such as beetles and flies, to spiders and lizards such as geckos. Independent of its systemic position and even the scale of the animal, we find surprising similarities related to the shape of the terminal elements (setal tips) in all these cases always have a similar thin shape as shown in Figure 1. Basically, all the spatulae are flattened and stuck on a rough substrate, while the free ends are oriented in the distal direction [8]. In order to understand the origin of the super-adhesive properties of these systems and, later, to reproduce them, a variety of studies, starting from the pioneering contributions of Kendall in the sixties [9], have focused on the peeling mechanism, by which a tape detaches from the flat substrate where it is attached [3,4,5,6,7,8,10,11]. Despite the interesting results obtained in these studies, the majority rely on the assumption of perfectly flat substrates. However, in order to reproduce good adhesive properties at macroscales, surface roughness cannot be ignored. This study takes a first step towards including the effect of microscale roughness into the contact mechanics of thin layers. As depicted in Figure 1, our model is a simple, yet explicative model for the contact between a rigid rough surface and an elastic layer of finite thickness supported by a constant pressure.

It is important to observe that solving the contact mechanics problem between rough solids is challenging even when using the half-space assumption, which treats any characteristic length in the interface as small compared to the thickness of the contacting bodies. Historically, the so-called multiasperity models [12,13,14,15,16] have been the first attempt to resolve the contact between elastically deformable and microscopically rough surfaces: fundamentally, these methodologies consider the rough surfaces to be composed of asperities—with a certain distribution of heights and radii of curvature—which behave like independent Hertzian punches without any mutual interaction. To include the effect of elastic deformation on all scales, Persson developed a different approach [17,18] based on the assumption that the contact pressure probability distribution is governed by a diffusive process as the magnification at which we observe the interface is increased. The theory is formulated in such a way that it is exact in full-contact conditions, while for partial contact, it provides an approximate solution predicting correctly, for example, a linearity between contact area and load (see, e.g., in [19]) and the logarithmic dependence of the interfacial separation on load (see, e.g., in [20]). Furthermore, a large variety of approaches have been developed to obtain quantitatively and qualitatively accurate results: these techniques include finite element methods (FEM) [21], boundary element methodologies (BEM) [22,23,24,25,26,27,28], molecular dynamics simulations [29,30,31] and hybrid approaches [32,33]. An important issue that these approaches have to deal with is their numerical convergence, which can be quite demanding due to the multi-scale nature of the problem.

Here, we extend the well-known and rather efficient Green’s function molecular dynamics (GFMD) technique [31] to the treatment of thin layers. The work is outlined as follows: Section 2 describes the mathematical formulation of the methodology, while results are presented and discussed in Section 3. Final remarks conclude the paper in Section 4.

## 2. Materials and Methods

We simulate a flat, elastic manifold of finite width that touches on one side a rigid, rough substrate fixed in space and, on the other side, a fluid reservoir, or, alternatively, a solid with negligible shear modulus in such a way that a uniform pressure is applied to the elastic layer (as shown in Figure 1). This is done using the GFMD method [31,34,35] as described in [36]; with Green’s functions [37] appropriate for our problem. The central aspects of the approach are described as follows. We denote the position of surface points facing the substrate by their lateral and normal coordinates, $r_{i}$, which are equally spaced on a two-dimensional surface, representing N=n×n grid points, *n* being typically 4096. In-plane periodic boundary conditions are employed and the system is treated as being homogeneous within the plane. The coordinate system is chosen with the highest point of the substrate set to zero. The (undeformed) elastic manifold touches the substrate in just this one point, when we apply an infinitesimally small force. The mean displacement at this load is said to be zero. Contact is complete when all points of the manifold touch the substrate. The displacement at that point is normalized to one.

The grid points are propagated in time according to Newton’s equation of motion, which is achieved with the Verlet algorithm. The total normal force, $F_{i}$, acting on a grid point is the sum of an external force, the elastic force, a damping force, and the (unknown) constraint force preventing the rigid surface from penetrating the thin layer. The external force is simply the external/fluid pressure $\sigma_{0}$ times the area element $\Delta A=a^{2}$ associated with the grid point. The elastic restoring on each atom is computed from the Fourier transform of the stress in Fourier space. The latter is obtained as

(1) $$\tilde{\sigma }\left(q\right)=\frac{1}{G(q)}\tilde{z}\left(q\right),$$

where the coefficients $\tilde{z}\left(q\right)$ are the Fourier transform of the grid point normal to the surface positions $z(r_{i})$ and q are in-plane wavevectors. For the given system, marked by a width *w* , the Fourier transform of the Green’s function is

(2) $$G\left(q\right)=\frac{2}{qE^{*}}f\left(qw\right)$$

where $E^{*}$ is the contact modulus of the elastic manifold and a finite-width correction factor [37] of

(3) $$f\left(t\right)=\frac{\sinh(2t)+2t}{\cosh\left(2t\right)-1-2t^{2}},$$

where t=qw is a dimensionless width. We note in passing that f(t) tends to one for t≫1 and scales as $t^{-3}$ for t≪1. This is, indeed, consistent with the equation governing thin plates (see [38,39] for more details).

The damping force is linear in particle velocity, or, when dynamics are solved in Fourier space, linear in the velocity of a given mode. The proportionality prefactor is best chosen such that the slowest mode of the system is slightly underdamped. For medium loads, it often turns out to be proportional to the load. The time step should be chosen as large as possible before dynamics become unstable, since we only attempt to identify mechanical equilibrium rather than true dynamics.

The constraint is established by setting each material point exactly onto the substrate if $z_{i}<h\left(r_{i}\right)$ after the Verlet time step, where h(r) denotes the height of the substrate. It is generated from its Fourier transform, which are complex random numbers with the following expectation values

(4) $$\begin{array}{ccc} \langle \tilde{h}\left(q\right)\rangle & = & 0 \end{array}$$

(5) $$\begin{array}{ccc} \langle |\tilde{h}{\left(q\right)|}^{2}\rangle & \propto & C(q). \end{array}$$

Real and imaginary components of $\tilde{h}\left(q\right)$ are drawn independently from each other. To avoid rare but potential freak fluctuations, we do not draw from Gaussian but instead a uniform random number with zero mean and a second moment according to the target spectrum. The surface spectra satisfy

(6) $$C\left(q\right)=C\left(q_{r}\right)\times \left\{\begin{array}{cc} 1 & \mathrm{for}q<q_{r}\\ q^{-2(1+H)} & \mathrm{for}q_{r}<q<q_{s}\\ 0 & \mathrm{else}, \end{array}\right.$$

where $q_{r}=2\pi /\lambda_{r}$ and $q_{s}=2\pi /\lambda_{s}$ are the roll-off and the cut-off wavenumber, respectively.

**Figure 2:**
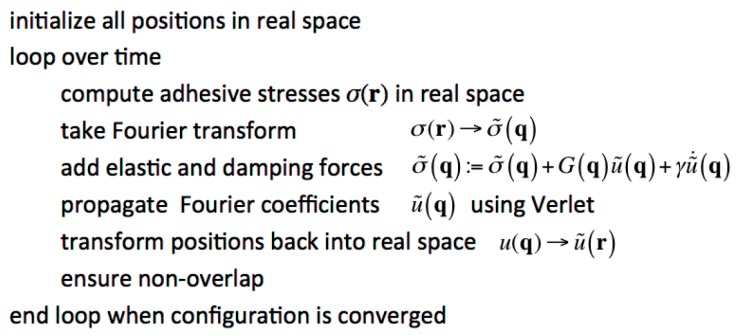
Schematic flow-sheet describing the Green’s function molecular dynamics (GFMD) method algorithm.

As proposed in [40], under the small-slope approximation method, we add an adhesive stress, which acts normal to the surfaces and, in real space, is equal to:

(7) $$\sigma_{\mathrm{adh}}\left(r\right)=\frac{\gamma_{0}}{\rho }\exp\left[-\left\{z\left(r\right)-h\left(r\right)\right\}/\rho \right],$$

where $\gamma_{0}$ is the energy gained per unit area when the two solids touch mechanically, z(r)−h(r) is the local gap between substrate and top solid, and *ρ* is the characteristic range of the attraction. We chose *ρ* sufficiently small so that adhesion can be characterized as short ranged. The implementation of the approach is summarized schematically in Figure 2.

The GFMD method can handle a very large amount of elements on a single node. This exceeds the size of most experimental surface topography measurements. Equilibration can be achieved within a few thousand time steps for most loads in most cases. The method only becomes inefficient for relative contact areas of less than 0.1% , in which case several hundred thousand iterations might be needed. Here, we only solve for static equilibrium. However, if the method was extended to dynamics, the accessible time span $T_{\mathrm{acc}}$ would depend on the level of discretization. As a rough rule of thumb, $T_{\mathrm{acc}}$ would be a few *μ*s times the distance between two grid points expressed in units of nanometers.

## 3. Results and Discussion

Results reported in this study relate to the contact between an elastic layer and a rigid fractal surface numerically generated by means of the spectral method as described in [31]. In particular, here we have generated fractal surfaces with $\lambda_{s}=4a$ for the short wavelength cut-off, $\lambda_{r}=64\lambda_{s}$ for the roll-off wavelength, and a system length of $8\lambda_{r}$. The Hurst roughness exponent *H* is equal to 0.8. Furthermore, we scale the surfaces in such a way that the root mean square gradient $\sqrt{<\nabla h^{2}>}$ is equal to 1.

**Figure 3:**
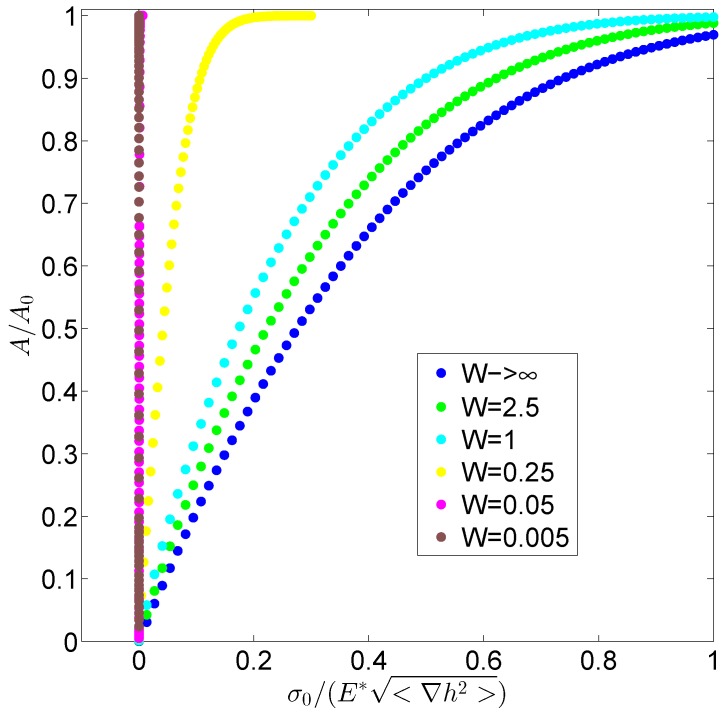
Contact area $A/A_{0}$ as a function of the dimensionless load $\sigma_{0}/\left(E^{*}\sqrt{<\nabla h^{2}>}\right)$ for different values of the ratio *W*.

Firstly, we focuse our attention on the contact mechanics between the rigid surface and an elastic layer with a negligible value of the surface adhesive energy *γ*: at this stage, our aim is to isolate the effect of the layer thickness and, specifically, how the contact properties change in comparison with thick substrates where the half-space assumption is valid. In Figure 3, we plot the relative contact area as a function of the dimensionless load $\sigma_{0}/\left(E^{*}\sqrt{<\nabla h^{2}>}\right)$ with $E^{*}$ being the composite Young modulus $E^{*}=1$ Pa and for different values of the dimensionless width $W=w/\lambda_{s}$ , where *w* and $\lambda_{s}$ are the layer width and the short wavelength cut-off, respectively. We observe that, by decreasing the ratio *W*, the contact compliance is increased: a normal load is fixed and a larger contact area is obtained. Indeed, as established by Persson’s theory [17,18] and generally accepted by the scientific community ([23,41]), for an half-space, the area/load relation is described by the equation $A/A_{0}=erf(k\sigma_{0}/(E^{*}\sqrt{<\nabla h^{2}>}$ with *k* being a proportionality coefficient that, when adhesion can be neglected, is approximately equal to k=2 [19,31]. When dealing with thin layers, we observe an increase of such a coefficient of several orders of magnitude.

**Figure 4:**
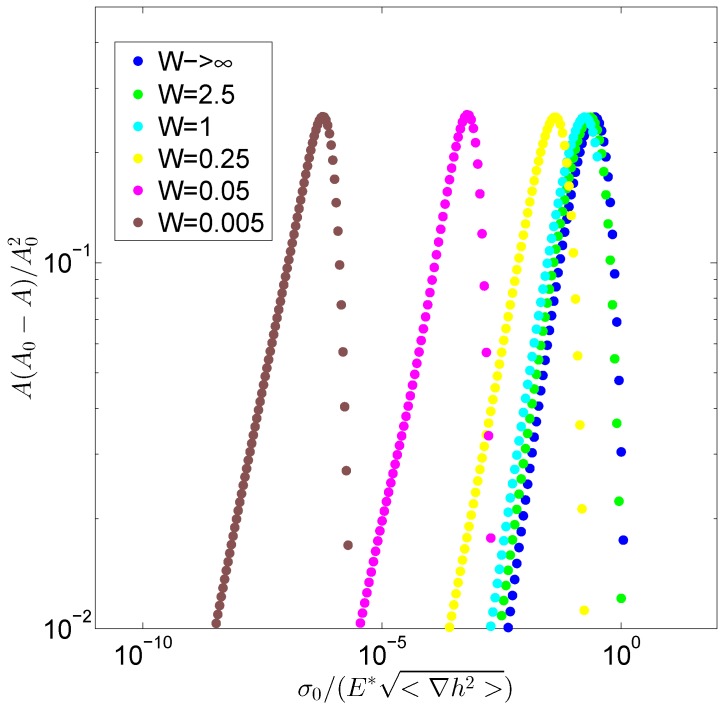
The quantity $A\left(A_{0}-A\right)/A_{0}^{2}$ is plotted against the dimensionless load $\sigma_{0}/\left(E^{*}\sqrt{<\nabla h^{2}>}\right)$ for different values of the ratio *W*.

In more detail, in order to better understand the role played by the thickness, in Figure 4 in a log-log plot, we report, again for different values of *W*, the quantity $A\left(A_{0}-A\right)/A_{0}^{2}$ as a function of the load, thus showing that the influence of a finite value of the layer width appears also with very low loads. Indeed, this entails a marked difference of the system under investigation (i.e., an elastic layer sustained by a constant pressure), not only in comparison with the classic half-space regime but also with other types of boundary conditions and, in particular, with the case reported in [42], where the deformable layer is bonded to a rigid half-space. In this last case, at low values of the contact area, the system behaves like an half-space and, only when the contact load is increased, we obtain a transition to a different regime where the thickness acquires an important role. This is consistent with the cut-off effect produced by a corrective coefficient Θ(qw), the main effect of which is to remove the low frequency contribution to the contact solution [42]. In contrast, under the conditions we investigate here, also at very low loads, the system shows a marked different trend and, in particular, the contact compliance results increased. This is due to the different mathematical form of the corrective coefficient f(qw) and may have important implications in many systems, including biological membranes and human skin, where very large contact areas may be produced with relatively low loads.

**Figure 5:**
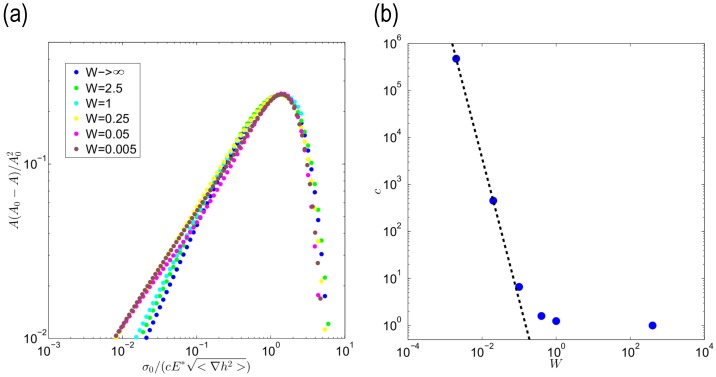
The quantity $A\left(A_{0}-A\right)/A_{0}^{2}$ is plotted against the dimensionless load multiplied for a correction factor $\sigma_{0}/\left(c\;E^{*}\sqrt{<\nabla h^{2}>}\right)$ (**a**). The correction factor *c* is plotted as a function of the width *W* (**b**). The dotted line refers to the asymptotic trend $∼W^{-3}$.

To appreciate the quantitative importance of a correct estimation of the finite thickness effects, in Figure 5a, we collapse the curves representing $A\left(A_{0}-A\right)/A_{0}^{2}$ into a master curve coinciding with the half-space solution by dividing the load for a corrective coefficient *c* that will be, as expected, a function of the dimensionless width *W*. Consequently, for this kind of system, we can obtain the following generalized area/load relation:

(8) $$\frac{A}{A_{0}}=\frac{1}{c\left(W\right)}{\left(\frac{A}{A_{0}}\right)}_{HS}$$

Indeed, beyond some differences for small contact area, the curves show a good overlap in Figure 5a. At the same time, we observe that the corrective coefficient *c* has a variation range spanning several orders of magnitude (see Figure 5b): this dramatically illuminates how important it is to account for the actual width of the layer. Furthermore, if we observe the asymptotic trend for very small values of *W*, we notice that $c\left(W\right)∼W^{-3}$ as expected given the form of the Green’s function coefficient f(qw).

**Figure 6:**
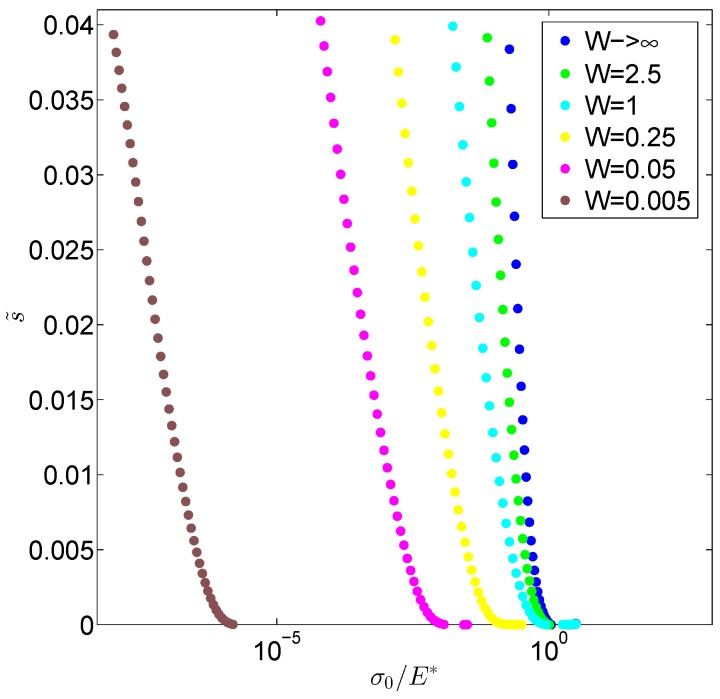
The dimensionless separation $\tilde{s}$ versus the load $\sigma_{0}/E^{*}$.

The increased compliance of the system can be noticed also when looking at the displacement averaged in the contact area as a function of the load. In detail, as shown in Figure 6, at relatively high loads, in agreement with many theoretical and numerical predictions [17,19,43], a logarithmic dependence between the quantity $\tilde{s}=(1-<u>/u_{\max})$ and $\sigma_{0}/E^{*}$ is found; as expected when running numerical simulation, at smaller loads, such a logarithmic trend is lost due the finiteness of the rigid surface employed in the computations. Indeed, we notice that, when the thickness *W* is reduced, fixed the load $\sigma_{0}/E^{*}$, we obtain a much smaller separation $\tilde{s}$ , consistently with a system that is more compliant.

Results shown so far refer to a case with negligible adhesion; however, the contact solution also shows a similar trend when adhesive effects are accounted for. Indeed, such a behavior is directly correlated with the elastic energy stored in the deformable layer. In our analysis, the dominant contribution to the system deformation is assumed to be due to the bending and, indeed, for W<<1, the mechanics of our layers coincides with that of thin plates [39]. The convention implies that any contribution related to the stretching energy is neglected: such an approximation is fully justified in a large variety of conditions, including, for example, the case of adhesive contact of insects and geckos [38].

**Figure 7:**
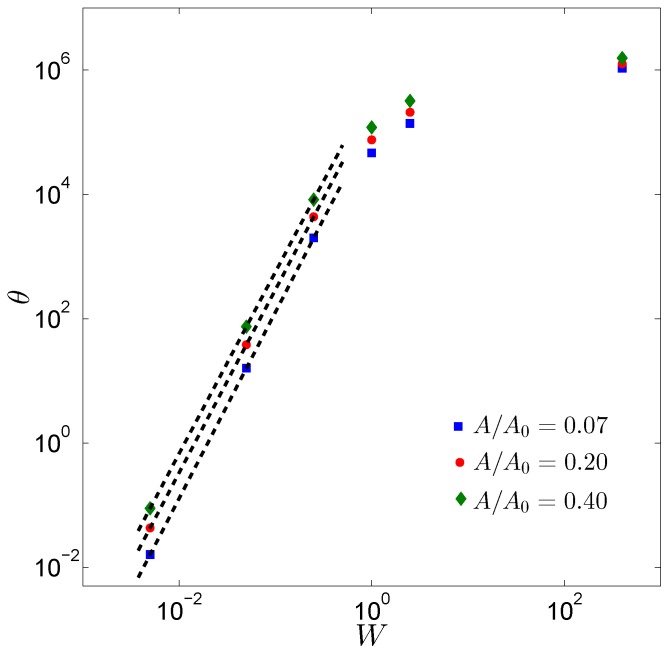
The adhesion parameter θ is plotted as a function of the dimensionless width *W* for three different values of $A/A_{0}$. The dotted lines refers to the asymptotic trend $∼W^{3}$.

Elastic energy stored in the layer is, indeed, a crucial quantity that determines the adhesive performance of the structure. More specifically, we define the parameter *θ* as $\theta =U_{el}/U_{ad}$ with $U_{el}$ and $U_{ad}$ equal to the elastic and to the adhesion energy, respectively. This is a qualitative measure of the competition between the elastic energy, which has to be stored to deform the contacting bodies, and the adhesive term, which corresponds to the bodies inclination to come into contact and create contact areas: the smaller *θ* is, the easier it is to obtain large contacts results. Consequently, *θ* can be considered a good way to estimate the adhesion capability. In Figure 7, for γ=5 $10^{-4}$ and μ=2, and for three different values of the contact area, *θ* dramatically decreases with *W*. This demonstrates that, indeed, thin layers are preferred to thick structures when good adhesion properties are desired due to the reduced compliance. Interestingly, as expected, for small values of *W*, *θ* scales again as $∼W^{3}$.

It is readily argued that the results we have just obtained are consistent with Persson’s contact mechanics theory [17]. Persson predicts that the contact area follows $erf(\sigma_{0}/\Delta \sigma \sqrt{2})$, where $\sigma_{0}$ is the apparent contact pressure perceived macroscopically, while Δσ can be interpreted as the width of the pressure distribution within the microscopically fully resolved contact points. This relation does not depend on the thickness of the elastic manifold. However, the layer width would affect the degree of broadening of the pressure distribution within the contact. Specifically, according to Persson theory, adding ΔC(q) to the height spectrum at wavevector q leads to an increase of $\Delta \sigma^{2}$ that is proportional to G(q)ΔC(q). Adding up all roughness would then lead to a value of $\Delta \sigma^{2}$ scaling proportionally to ${\left(t^{3}E^{*}\right)}^{2}\langle \nabla h^{2}\rangle$ (see also, for example, Equation (31) in [36]). For self-affine rough surfaces with a Hurst exponent 0<H<1, the mean-square surface-height gradient is dominated by the short-wavelength terms. For truly thin substrates resting on a fluid foundation, the integrated broadening Δσ therefore scales as ${\left(t/\lambda_{s}\right)}^{3}$ times the Green’s function of the semi-infinite manifold, as we descrived in Section 2, and as is revealed in our numerical results.

## 4. Conclusions

In the present study, we develop a numerical methodology based on the GFMD to study the contact between a rigid fractal surface and an elastic layer sustained by a uniformly applied pressure. Indeed, such a scheme provides a model for a large variety of biological systems, spanning from the human skin to biological membranes. All these cases can be studied by the GFMD method that has been modified multiplying the Green’s function G(q) for a corrective parameter *f* that is related to the dimensionless width t=qw. Our results show that, in comparison with solids with large values of the thickness, where the half space approximation may be adopted, thin layers show a much larger compliance that marks both the contact area and the separation when analyzed as functions of the load. In particular, when looking at the contact area, we observe that the classic area/load relation can be corrected by introducing a coefficient *c*. Interestingly, *c* scales as $W^{-3}$ with *W* being the dimensionless width of the layer: this is related to the mathematical form of the the Green’s function corrective coefficient f(qw) and is consistent with Persson’s contact theory. Ultimately, we show why thin layers should be preferred when good adhesive properties and large contact areas are desired.
